# Analysis of the Response Speed of Musculature of the Knee in Professional Male and Female Volleyball Players

**DOI:** 10.1155/2014/239708

**Published:** 2014-06-09

**Authors:** D. Rodríguez-Ruiz, I. Diez-Vega, D. Rodríguez-Matoso, M. Fernandez-del-Valle, R. Sagastume, J. J. Molina

**Affiliations:** ^1^University of Las Palmas de Gran Canaria, Campus Universitario de Tafira, s/n, Edificio de Ciencias de la Actividad Física y el Deporte, 35017 Las Palmas de Gran Canaria, Spain; ^2^European University of Madrid, Calle Rio Tajo s/n, Villaviciosa de Odón, 28670 Madrid, Spain; ^3^Department of Health, Exercise, and Sport Sciences, Texas Tech University, P.O. Box 43011, Lubbock, TX 79409, USA; ^4^University of País Vasco, Carretera Lasarte s/n, 01007 Vitoria-Gasteiz, Spain

## Abstract

The aim of this study was to evaluate the normalized response speed (Vrn) of the knee musculature (flexor and extensor) in high competitive level volleyball players using tensiomyography (TMG) and to analyze the muscular response of the vastus medialis (VM), rectus femoris (RF), vastus lateralis (VL), and biceps femoris (BF) in accordance with the specific position they play in their teams. One hundred and sixty-six players (83 women and 83 men) were evaluated. They belonged to eight teams in the Spanish women's superleague and eight in the Spanish men's superleague. The use of Vrn allows avoiding possible sample imbalances due to anatomical and functional differences and demands. We found differences between Vrn in each of the muscles responsible for extension (VM, RF, and VL) and flexion (BF) regardless of the sex. Normalized response speed differences seem to be larger in setters, liberos and outside players compared to middle blockers and larger in males when compared to females. These results of Vrn might respond to the differences in the physical and technical demands of each specific position, showing an improved balance response of the knee extensor and flexor musculature in male professional volleyball players.

## 1. Introduction

Volleyball is a sport in which players frequently perform technical actions which, from the mechanical perspective, involve a flexion-extension of the ankle, knee, and hip joints. The efficacy of the technical action depends to a great extent on the effectiveness of this movement in actions involving displacement and/or jumping.

The development of such movements depends on the skill of the athletes, while their efficacy depends on technical (spike or block), practical (jump or landing), and conditional (physical abilities) factors. The learning and automation process which the athlete has followed during his or her training is likewise decisive. In this regard, specialisation in order to achieve a high level of performance in a sporting pursuit involves the standardisation of training methods so as to bring about structural and neural adaptations properly aligned with the inherent demands of the activity to be performed and the position assigned to the player.

The morphological, muscular, and conditional differences between the male and female players occupying particular different positions may become qualitatively distinctive variables in their performance and predisposition to injury. In this regard, the greater activation of the ischiotibial musculature observed in landing after a jump and/or the coactivation which occurs between the flexor and extensor musculature of the knee joint when jumping or changing direction [1–9] are the main reasons which may cause an athlete to suffer an injury or fail to achieve the desired performance.

It is therefore necessary to perform a highly precise, individualized and localized evaluation of those muscular structures which are most commonly employed when playing volleyball. In accordance with the specific demands of male and female players and the particular position in which they play, since this will represent a conditional burden and a different technical demand [10–13] which will affect the flexor and extensor muscles of the knee joint in different ways.

In monitoring the muscular activation of the groups involved, tensiomyography (TMG) serves as a noninvasive diagnostic method which requires no effort on the part of the subject in question and which can be particularly useful to assess the rigidity, mechanical characteristics, and contractile capacity of superficial muscles when activated by an electrical stimulus of controlled intensity [14–27]. Meanwhile, in previous studies TMG has proven itself to be a sensitive, reliable, and methodologically valid tool to assess relevant differences of each specific player position, the sport skills, and the position on court [25]. In addition, Rodríguez-Ruiz et al. [26] reported sex differences in the normalized response speed (Vrn) in the musculature responsible for extension and flexion of the knee joint. The use of Vrn allows avoiding possible sample imbalances due to anatomical and functional differences and demands. For example, Rodríguez-Ruiz et al. [27] found differences for Vastus Lateralis but not for Biceps Femoris between subjects of different ages and levels of physical activity.

Based on these contributions [26, 27], the aim of this study was to employ TMG as the instrument for evaluation of the normalized response speed (Vrn) of the flexor and extensor musculature of the knee in high competitive volleyball players and to analyze the muscular response of the vastus medialis (VM), rectus femoris (RF), vastus lateralis (VL), and biceps femoris (BF) in accordance with the specific position they play in their teams.

## 2. Method

### 2.1. Participants

One hundred sixty-six players were evaluated: 83 females (23.9 years ± 5.1, 178.1 cm ± 7.3, and 71.9 Kg ± 8.9) and 83 males (25.9 years ± 4.8, 191.3 cm ± 7.4, and 87.7 Kg ± 9.3). They belonged to eight teams in the Spanish Women's Superleague and eight in the Spanish Men's Superleague. More than 75% of the players had at some point represented their country in international matches either at senior or junior levels.

All participants were informed of the potential risks associated with the study and signed written consent forms, approved in advance by the University of Las Palmas de Gran Canaria's Research Ethics Committee, in accordance with the guidelines set out in the Declaration of Helsinki on research involving human subjects (adopted by the 18th Assembly of the World Medical Association, held in Helsinki in 1964, and amended by the 59th General Assembly, held in Seoul in 2008) which were strictly followed by all members of the research team.

### 2.2. Measurement Procedure

The muscles analysed by means of TMG were the vastus medialis (VM), rectus femoris (RF), vastus lateralis (VL), and biceps femoris (BF), based on the premise that these are the most significant in the technical actions involved in specific movements and jumping [28].

A pressure sensor was attached to the belly of the selected muscle, ensuring that it was positioned perpendicular to the muscle belly [29] and measurements were performed supine for VL, VM, and RF and prone (BF) both set at 30° angle of knee flexion [30–33]. In order to provoke contraction a bipolar electrical current was applied. One single intensity (110 mA) of one millisecond in duration was applied, via two electrodes positioned at 5 cm the proximal and distal to measurement point in such a way as not to affect the tendons [34]. A pause between electrical stimuli was set in order to avoid the phenomenon of posttetanic activation [34, 35]. The reproducibility of the method and the validity of the experimental protocol employed by TMG have been studied in a number of works, revealing this to be a highly precise tool [19, 26, 31, 34, 36–39].

Evaluation of the desired muscle provides numerical information as to the magnitude of the radial displacements of the transversal muscle fibres and the moment when these occur [29, 34]. Out of all the data obtained, we focus on the study of the* normalized response speed* (Vrn). This parameter was used to compare gender mechanical muscles differences on high performance volleyball players [26] and subjects with different age [27].

The* normalized response speed *(Vrn) represents the relationship between the difference in the radial displacement between 10% and 90% of muscle belly maximum radial displacement (Dm) and the increase in the muscular contraction time between these same values. Valenčič and Knez [29] state that in order to be able to compare the values obtained in different muscles this increase in time must be normalized. This is achieved by dividing the equation previously performed by the Dm for each muscle. The authors do not state that the increase in the displacement between 10% and 90% of Dm is equal to 0.8 per Dm. As a result, the normalized speed of response would be equal to 0.8 divided by the increase in the muscular contraction time between 10% and 90% of Dm. The data obtained for the left leg and the right leg were added together in order to be able to illustrate differences without differences of lateral symmetry influencing the final functional analysis [26].

We consider it necessary to normalize the data obtained from the difference in radial displacement of a muscle belly between 10% and 90% of the maximal amplitude of the muscle response, because of the differences found in Dm and contraction time for different muscles. The calculation of Vrn allows us to isolate the interference that we can find due to the individual characteristics of each subject of the sample as well as from the anatomical differences and the different functional demands of the musculature responsible for extension (VM, RF, and VL) and flexion (BF). This calculation has already been used in previous studies, finding clear differences terms of muscle response between male and female volleyball players: women displayed a more pronounced difference in the Vrn of the musculature responsible for extension (vastus medialis, rectus femoris, and vastus lateralis) and flexion (biceps femoris) of the knee joint than men [26] and, in a subsequent study differences were found for vastus lateralis but not for biceps femoris between subjects of different ages and levels of physical activity [27], demonstrating that Vrn is a parameter which allows us to isolate these anatomical and functional interferences.

### 2.3. Statistical Analysis

All data were checked for normality using the Kolmogorov-Smirnov, where normality was assumed if *P* > 0.05. A 2 × 4 × 5 mixed ANOVA was used to analyze the interactions between sex (male, female) or specific position (setter, middle blocker, outside hitter, or opposite hitter) with the musculature (BF, RF, VM, and VL). Degrees of freedom were corrected using Greenhouse-Geisser estimates. Bonferroni test was used for post hoc analyses. All the statistics were performed using the SPSS-v21 statistical package (SPSS Inc., Chicago, IL, USA), and with an alpha significance of 0.05.

## 3. Results

An interaction within subjects was shown for all muscles (*F*(1.92) = 35.40, *P* < 0.001, *η* ^2^ = 0.23). In addition, the differences between muscles remained present when allocated by sex (*F*(1.92) = 15.79, *P* < 0.001, *η* ^2^ = 0.10). Nevertheless, no interactions were found when allocated by specific position (*F*(7.70) = 1.32, *P* = 0.24, *η* ^2^ = 0.03) or by sex and specific position (*F*(7.70) = 0.75, *P* = 0.64, *η* ^2^ = 0.02).

**Figure 1:**
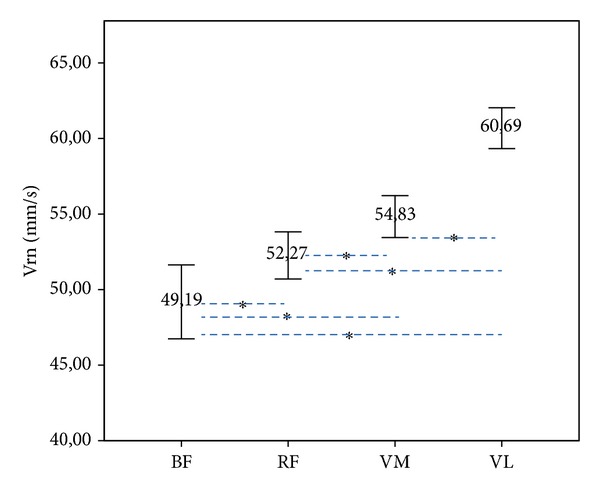
Differences in the mean normalized response speed (Vrn) in mm/s in biceps femoris (BF), rectus femoris (RF), vastus medialis (VM), and vastus lateralis (VL), in both male and female players (*P ≤ 0.05) (95% of the confidence interval (IC)).

Normalized response speed (Vrn) was significantly different between all of them. BF has shown lower Vrn compared to RF (*P* = 0.022), VM (*P* < 0.001), and VL (*P* < 0.001). In addition, RF has shown lower Vrn than VM (*P* = 0.012) and VL (*P* < 0.001), and VM has shown lower Vrn than VL (*P* < 0.001) (Figure 1).

**Figure 2:**
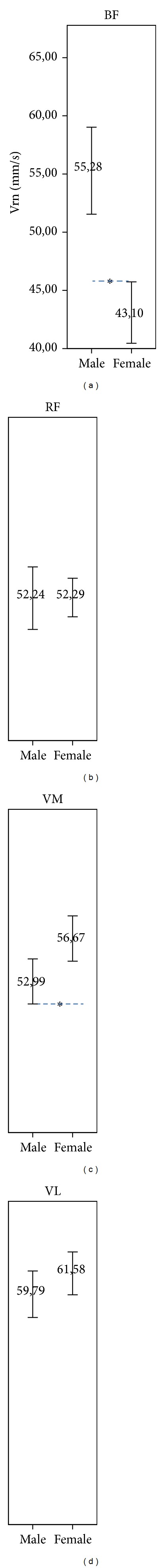
Sex differences in mean normalized response speed (Vrn) in mm/s in biceps femoris (BF), rectus femoris (RF), vastus medialis (VM), and vastus lateralis (VL), comparing male and female players (*P ≤ 0.05) (95% of the confidence interval (IC)).

Significant differences depending on sex were found in BF (*F*(1) = 18.57, *P* < 0.001, *η* ^2^ = 0.12) and VM (*F*(1) = 6.30, *P* = 0.01, *η* ^2^ = 0.04), but not for RF (*F*(1) = 0.00, *P* = 0.99, *η* ^2^ < 0.01) or VL (*F*(1) = 1.77, *P* = 0.19, *η* ^2^ = 0.01). Vrn in males was greater than in females in BF (*P* < 0.001) but lower in VM (*P* = 0.013) (Figure 2).

**Figure 3:**
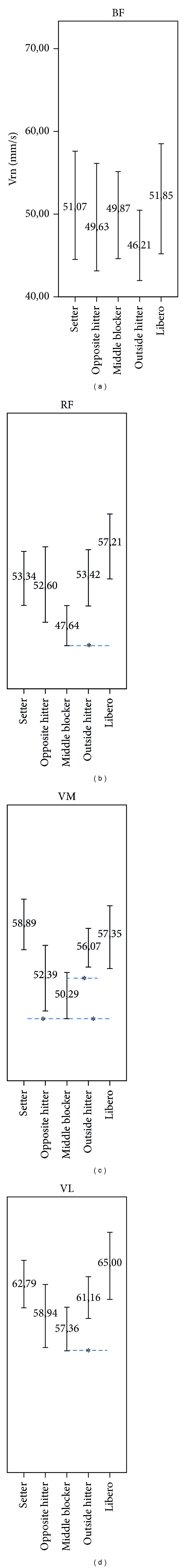
Differences in mean normalized response speed (Vrn) in mm/s depending on the specific position (setter, opposite hitter, middle blocker, outside hitter, and libero) in biceps femoris (BF), rectus femoris (RF), vastus medialis (VM), and vastus lateralis (VL), in both male and female players by team position (*P ≤ 0.05) (95% of the confidence interval (IC)).

No interactions (muscle × position) were found. When post hoc analyses were applied, no differences were found in BF (*F*(4) = 0.48, *P* = 0.75, *η* ^2^ < 0.01). However, significant differences were found in RF (*F*(4) = 3.78, *P* = 0.006, *η* ^2^ = 0.10), VM (*F*(4) = 5.87, *P* < 0.001, *η* ^2^ = 0.15), and VL (*F*(4) = 3.64, *P* = 0.007, *η* ^2^ = 0.09) for all positions. Vrn in middle blockers was lower in VM compared to setters (*P* < 0.001), outside hitters (*P* = 0.023), and liberos (*P* = 0.021). Middle blockers also showed lower Vrn in RF (*P* = 0.006) and VL (*P* = 0.010) (Figure 3).

A main effect was shown for muscle × position (*F*(4) = 4.29, *P* = 0.003, *η* ^2^ = 0.11), not for muscle × sex (*F*(1) = 1.34, *P* = 0.25, *η* ^2^ < 0.01) or sex × position (*F*(4) = 0.76, *P* = 0.55, *η* ^2^ = 0.02).

**Figure 4:**
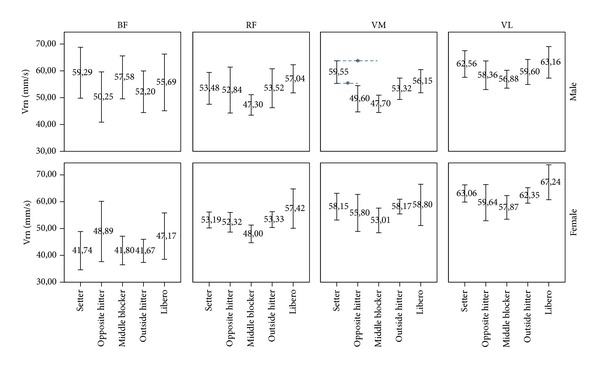
Mean normalized response speed (Vrn) differences in biceps femoris (BF), rectus femoris (RF), vastus medialis (VM), and vastus lateralis (VL) depending on sex and position (*P ≤ 0.05) (95% of the confidence interval (IC)).

When Vrn differences were analyzed depending on sex and position, significant differences were found in VM for males (*F*(4) = 5.66, *P* < 0.001, *η* ^2^ = 0.15), where Vrm was greater in setters when compared to opposite hitters (*P* = 0.025) and middle blockers (*P* < 0.001). No other differences were found (Figure 4).

**Figure 5:**
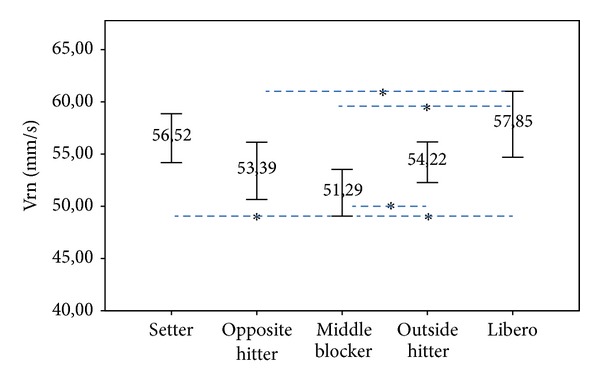
Main effect of mean normalized response speed (Vrn) in mm/s compared by team position (*P ≤ 0.05) (95% of the confidence interval (IC)).

When specific positions were analyzed, middle blockers showed the lowest Vrn (51.27 ± 1.04 mm/ms; *P* = 0.002), followed by the opposite hitters (53.46 ± 1.53 mm/ms; *P* = 0.035), and outside hitters (54.27 ± 1.53 mm/ms; *P* < 0.001). The volleyball players with greater Vrn were the liberos (56.38 ± 1.20 mm/ms; *P* = 0.045) and setters (56.38 ± 1.53 mm/ms; *P* = 0.050) (Figure 5).

## 4. Discussion

In this study we found evidence of differences in terms of the speed of response of the musculature responsible for extension (VM, RF, and VL) and flexion (BF) in the knee joint of professional male and female volleyball players (Figure 1).

The adipose tissue arises as an important factor affecting the muscular responses to electrical stimulation. In this sense, Diez-Vega et al. [40] analyzed the correlation of the values of fat percentage (%*F*), front thigh skinfold (FTS), and thigh girth (TG) to the maximal radial deformation (Dm) of the rectus femoris (RF) obtained using TMG. Results did not show differences between samples (FTS versus Dm: *r* ^2^ = 0.0096; *P* = 0.19, and %*G* versus Dm: *r* ^2^ < 0.001; *P* = 0.98), neither when TG was used as a control variable (FTS versus Dm: *r* ^2^ = 0.01; *P* = 0.18, and %*G* versus Dm: *r* ^2^ < 0.001; *P* = 0.82). These results point out that Dm is not affected by body composition variables such as %*F*, FTS, or TG in high performance volleyball players.

The male players, meanwhile, revealed a greater balance in the response of the extensor and flexor musculature of the knee. Previous studies explain this behaviour as a result of the difference in activation of the musculature involved, indicating that women, with a reduced knee flexion, perform the jumping action mainly by calling on the quadriceps, whereas men perform this movement with a more intense activation of the quadriceps and isquiotibiales [2, 6, 8, 26]. These considerations are reinforced in the results found on comparing the Vrn results between men and women, it being observed that Vrn in males was greater in males than in females for BF (*P* < 0.001), but lower in VM (*P* = 0.013) (Figure 2). In this sense, Ebben et al. [41] reported that, during the precontact phase of the jump, males activate quadriceps muscle earlier than females and that males are hamstring's activity dominate during the postcontact phase compared to females.

Results such as the Vrn differences between sex and BF or VM (Vrn greater in males compared to females) are supported by Rodríguez-Ruiz et al. [26]. This study suggests that morphological differences may be caused by sex differences. The shape and proportions of the pelvis, as well as the position of the bony structures of the lower limb, are especially important in this sport. Hip width and external tibia rotation prompt flexion-extension of the knee, leading to a higher probability of injury amongst women [42–44]. Further, Rodríguez-Ruiz et al. [26] argue that these structural differences, combined with differences in mechanical response (Vrn) of the RF, VL, VM, and BF, indicate the possible development of a certain instability in the knee joint, perhaps adversely affecting the application of forces in the jump [8] and increasing the risk of injury on landing [1, 3–5, 7, 9].

Several anatomic and histochemical investigations [45–50] revealed two additional but always dissectible parts of the vastus medialis (VM) muscle: the vastus medialis obliquus (VMO) and the vastus medialis longus (VML). VMO runs into the quadriceps tendon, the superomedial quadrant of the patella, and the anterior medial capsule. The fibers in this short head are more horizontal, deviating an average of 50°–55° medially from the femoral axis. They have a specific role in the alignment and, therefore, an important role at the end of the extension as they lock the muscle of the knee. The long head inserted more proximally into the base of the patella is described as VML. The fibers in this long head are directed vertically, deviating an average of 15°–18° medially from the longitudinal axis of the femur. VML is purely an extensor of the leg. VMO is a weak extensor of the leg, but it plays an important role in keeping the patella on track in gliding on the femoral condyles. The medially directed forces of the VMO counteract laterally directed forces of the vastus lateralis, thus preventing lateral displacement of the patella in the trochlear groove [47]. Increased values for VM in females might suggest that this muscle plays an important role in the knee stabilization, but further investigation is needed in others to confirm such hypothesis.

An analysis of the results by specific position reveals both in men and in women (Figure 3) that there are variations in the muscular speed of response in accordance with the physical and technical demands of each playing position. These data coincide with those found by Rodríguez-Ruiz et al. [25] for male and female beach volleyball players, it being found that TMG proved itself to be a tool sensitive to the changes produced by muscular adaptations to technical actions in the competitive context. In fact, the middle blockers are the players revealing the greatest differences with regard to the other specific positions (Figure 3). It is among these players that we find the greatest Vrn in BF, and the lowest in VM and RF, as opposed to the situation with other players, where the highest value corresponds to VL and the lowest has the greatest variations between the positions (Figure 3). This behaviour can be explained if we take into consideration the fact that the action specific to the middle blockers is that of blocking and that in one single rally point they may perform various jumps or perform some other explosive action after receiving a jump. This same behaviour occurs with the setters who after a controlled jump (suspended set) generally perform another explosive action. This combines with the fact that in both positions and in the liberos there is a large number of lateral movements which end with flexion of the knee joint in order to balance the body and provide a secure platform for the subsequent technical action. Outside and opposite hitters perform greater amount of movements (different techniques) with increased knee flexion. In the case of the setters and liberos the knee flexion is related to specific movements such as controlled jumps (setters) or a large number of lateral movements ended in knee flexion (liberos). This is the reason why the setters and the liberos also reveal high values in BF (Figure 3). Sheppard et al. [51] reported differences in the ability to tolerate high stretch loads depending on the specific position, as in the depth jump, where outside and opposite players bear the major loads compared to setters, middle blockers, and liberos. In this sense, there are studies listed in the specialist bibliography demonstrating that employment of a prior activity which positively conditions the subsequent action of muscular contraction will increase the vertical jump [52, 53]. This would lead us to suppose that this jump prior to an explosive action, whether in setting, in dummying a shot or jump to the opposing middle blocker and a subsequent move to the wing by the middle blocker could lead to a state of complete potentiation [54] in the rapid contraction motor units, increasing the* muscular speed of response *to a contraction generated by an external electrical stimulus in BF.

Figure 3 reveals that the middle blockers are the players with the lowest RF values in comparison with other positions; this being in fact statistically significant in the liberos (*P* = 0.006), the players with the greatest speed of response. These differences in RF could be the result of the position of the body when performing the specific technical actions of the position, as the middle blockers are always close to the net and the body position always has a high centre of gravity, whereas the liberos are in the* second line*, further away from the net with a greater variation in the flexion extension of the hip when they perform defensive actions, with a lower centre of gravity.

When Vrn differences were analyzed depending on sex and position, significant differences were found in VM for males, where Vrm was greater in setters when compared to opposite hitters (*P* = 0.025) and middle blockers (*P* < 0.001). No other differences were found (Figure 4). Perhaps, these values for VM in setters might suggest that this muscle plays an important role in the knee stabilization, but further investigation is needed in others to confirm such hypothesis. Sheppard et al. [55] also found differences on the physiological demands and characteristics, and the jumping ability depending on the specific position in elite male volleyball players.

Finally, the results between muscles by position are when specific positions were analyzed (Figure 5): middle blockers showed the lower Vrn (51.27 ± 1.04 mm/ms; *P* = 0.002), followed by the opposite hitters (53.46 ± 1.53 mm/ms; *P* = 0.035), and outside hitters (54.27 ± 1.53 mm/ms; *P* < 0.001). The volleyball players with greater Vrn were the liberos (56.38 ± 1.20 mm/ms; *P* = 0.045) and setters (56.38 ± 1.53 mm/ms; *P* = 0.050).

### 4.1. Future Studies

We believe it is necessary to continue studying the results provided by TMG, comparing these with height of jump, anthropometric measurements, and age and race of the players. These studies will provide a better understanding of sex differences that will allow developing the most appropriate training strategies for knee injuries and muscular imbalances in females.

## 5. Conclusions

Differences between Vrn in the musculature responsible for extension (VM, RF, and VL) and flexion (BF) of the knee are present in professional volleyball players regardless of sex. Those differences seem to be larger in setters, liberos and outside players compared to middle blockers and larger in males when compared to females.

These results of Vrn might respond to the differences in the physical and technical demands of each specific position, showing an improved balance response of the knee extensor and flexor musculature in male professional volleyball players.
